# An ambient air quality evaluation model based on improved evidence theory

**DOI:** 10.1038/s41598-022-09344-0

**Published:** 2022-04-06

**Authors:** Qiao Sun, Tong Zhang, Xinyang Wang, Weiwei Lin, Simon Fong, Zhibo Chen, Fu Xu, Ling Wu

**Affiliations:** 1 School of Information Science and Technology, Beijing Forestry University, Beijing, 100083 China; 2 Engineering Research Center for Forestry-Oriented Intelligent Information Processing of National Forestry and Grassland Administration, Beijing, 100083 China; 3 School of Computer Science and Engineering, South China University of Technology, Guangzhou, 510006 China; 4 Department of Computer and Information Science, University of Macau, Taipa, Macau SAR

**Keywords:** Computer science, Information technology, Statistics, Environmental impact

## Abstract

It is significant to evaluate the air quality scientifically for the management of air pollution. As an air quality comprehensive evaluation problem, its uncertainty can be effectively addressed by the Dempster–Shafer (D–S) evidence theory. However, there is not enough research on air quality comprehensive assessment using D–S theory. Aiming at the counterintuitive fusion results of the D–S combination rule in the field of comprehensive decision, an improved evidence theory with evidence weight and evidence decision credibility (here namely DCre-Weight method) is proposed, and it is used to comprehensively evaluate air quality. First, this method determines the weights of evidence by the entropy weight method and introduces the decision credibility by calculating the dispersion of different evidence decisions. An algorithm case shows that the credibility of fusion results is improved and the uncertainty is well expressed. It can make reasonable fusion results and solve the problems of D–S. Then, the air quality evaluation model based on improved evidence theory (here namely the DCreWeight model) is proposed. Finally, according to the hourly air pollution data in Xi’an from June 1, 2014, to May 1, 2016, comparisons are made with the D–S, other improved methods of evidence theory, and a recent fuzzy synthetic evaluation method to validate the effectiveness of the model. Under the national AQCI standard, the MAE and RMSE of the DCreWeight model are 1.02 and 1.17. Under the national AQI standard, the DCreWeight model has the minimal MAE, RMSE, and maximal index of agreement, which validated the superiority of the DCreWeight model. Therefore, the DCreWeight model can comprehensively evaluate air quality. It can provide a scientific basis for relevant departments to prevent and control air pollution.

## Introduction

Due to the rapid development of industrialization and urbanization, large amounts of industrial pollutants are discharged, which has led to increasingly prominent environmental problems. Global air pollution is one of the most important environmental problems^1^, affecting people's productivity and health. Air pollution has become a great health hazard to human respiratory system, which can exacerbate asthma and chronic obstructive pulmonary disease^2^. Therefore, how to scientifically evaluate ambient air quality has become a research hotspot. It is beneficial to the implementation of pollution control by transportation or environmental management departments.

Many air quality evaluation methods have been proposed at home and abroad. It mainly includes air quality index (AQI), air quality composite index (AQCI), principal component analysis (PCA)^3^, gray clustering method^4,5^, and fuzzy synthetic evaluation (FSE)^6,7^. The national AQI method is widely used to assess the air quality level around the world. But it ignores the comprehensive effects of multiple pollutants^8^. The national AQCI considers the comprehensive acts of main pollutants on air quality. A high AQCI value means high pollution, but the specific degree of air pollution is not intuitionistic and clear. Li et al. analyzed the relationship between meteorological factors in Beijing using nonlinear regression and PCA analysis methods^9^. But it is not clear to obtain the air quality level. At present, FSE models have been proposed to comprehensively evaluate air quality. Lü et al. established the weight set of pollutants using the method of excessive times and comprehensively evaluated the air quality in the Beijing-Tianjin-Hebei region^10^ through the weighted FSE model. Zhang et al. comprehensively evaluated annual and quarterly air quality by the FSE method in Lanzhou City^11^. Based on the basic FSE models, Wang et al. proposed a secondary FSE model to evaluate the daily air quality in Caofeidian District, Tangshan City^12^. The above FSE models well addressed the ambiguity of air quality and quantified the comprehensive pollution degrees. However, it is subjective to evaluate the air quality by excessive times weighted method in the above FSE models. Li et al. proposed the entropy weight method to objectively evaluate air quality^13^, but the precision of evaluation was not high. Therefore, aiming at the above problems, this paper used the entropy weighted method and excessive times method to establish the combined weights of air pollutants.

The atmospheric environment is dynamic and complex. There are many uncertain factors in the process of environmental air quality assessment. Fortunately, evidence theory^14^ has the advantages in dealing with the ambiguity of air quality, and it is widely used in the comprehensive evaluation field^15,16^. Xia et al. evaluated the AQI level and predicted air quality using rough set and the D–S theory^17^. But it was not aimed at the evaluation of comprehensive air quality. In addition, D–S theory may make counterintuitive fusion results^18^ when pieces of evidence are highly conflicting. How to resolve high evidence conflict^19^ is the key issue.

Aiming at the above problems of D–S theory, a lot of work has been researched. Sun et al. introduced the evidence credibility and proposed a combination rule^20^ to distribute evidence conflicts. But the method ignored the weights of evidence, which affected its practical application. He Bing et al. classified evidence and combined each classification fusion result by the weighted mean method and D–S theory^21^, which avoided direct fusion of conflicting evidence. As the multi-criteria decision-making problem, Ma et al. pointed out that the final choice of the decision-maker may be adjusted and changed with the importance of evidence^22^. It can be analyzed that weights of evaluation factors are key to the final fusion results. Fei et al. determined the comprehensive weight based on the subjective weight method and objective entropy weight method to make the decision^23^.

At present, the above types of research on the evidence conflict are all measured according to the basic probability assignment (BPA) of the evidence. However, the measured evidence conflict is extremely sensitive to the changes of BPA. It is too dependent on subject BPA values to solve the fuzzy comprehensive evaluation problem effectively. Hence, this article proposed evidence decision credibility by calculating the dispersion of decision-makings, which can objectively measure evidence decisions conflict. In addition, the weights of evidence are objectively established by the entropy weight method. Although the improved evidence theory can fuse conflict evidence, there is not much research on the comprehensive air quality evaluation using improved evidence theory. Therefore, this paper proposed an air quality evaluation model based on improved evidence theory.

Specifically, the main contributions of this paper can be summarized as follows:

- A combination rule with evidence decision credibility and evidence weight is proposed in this paper. And a case validates its effectiveness. The counterintuitive problem of D–S theory is solved and the credibility of fusion results is improved using the proposed improved evidence theory.
- The air quality evaluation model (DCreWeight model) based on the improved evidence theory is proposed to evaluate air pollution situations comprehensively, which can effectively handle the uncertainty in comprehensive air quality evaluation.
- In the DCreWeight model, membership functions of the six air pollutants are built based on fuzzy theory. And they are transformed into BPA functions, which better deal with the ambiguous information of air quality levels.
- In the DCreWeight model, considering the contribution of different pollutant concentrations to air quality, the combined weights of air pollutants are established by subjective excessive times weight method and objective entropy weight method, which improves the accuracy of entropy weight method.
- Comparisons are made with the D–S, two improved methods of evidence theory and a recent FSE method. The results of air quality evaluation in Xi’an show that the DCreWeight model has the minimal MAE, RMSE, and maximal index of the agreement under the national AQI standard and AQCI standard, which is superior to the other methods.

The novelty of the proposed method is based on the improved evidence theory, which is complementary to the traditional air quality assessment methods. The rest paper is organized as follows: “Backgrounds” section presents the background of evidence theory. “Improved evidence theory” section presents the improved evidence combination rule. “Ambient air quality evaluation model” section establishes the model of the ambient air quality evaluation based on improved evidence theory. “Results” section is the application of air quality evaluation model in Xi’an. “Conclusion” section concludes the paper and advances some prospects.

## Backgrounds

In this section, to better understand the definitions in the subsequent content, the important nomenclature descriptions are listed in advance. Then the background of evidence theory is presented. The main nomenclature descriptions are as follows:

D–S theory: Dempster–Shafer evidence theory;
DCre-Weight algorithm: an improved evidence theory with evidence weight and evidence decision credibility;
DCreWeight model: air quality evaluation model based on improved evidence theory;
AQI: Air Quality Index;
AQCI: Air Quality Composite Index;
MAE: mean absolute error;
RMSE: root mean squared error;
AQI_an index of agreement: the proportion of the number of days when the evaluation result is equal to the AQI level;
FSE: fuzzy synthetic evaluation;
PCA: principal component analysis;
BPA: basic probability assignment function;
*K*: conflict coefficient of pieces of evidence.
$$\varepsilon$$
evidence credibility;
$$\overline{\varepsilon }$$
decision credibility;
*d*: standard deviation;
q: average evidence;
m(*A*): the basic probability assignment of set *A*;
*m*_1_$$\oplus$$*m*_2_…$$\oplus$$*m*_n_
the orthogonal sum of evidence;
w: weight matrix about the weights of pieces of evidence;
Hybrid-Rule: the combination of weight mean rule and D–S theory;
KCre-Sun: the combination rule with credibility based on average evidence conflict, proposed by Sun Quan et al.;
MFs: membership functions;
*U*: the set of evaluation objects.

### D–S evidence theory

If a set is defined as ${\Theta }$ and all elements in the set are independent and mutually exclusive, ${\Theta }$ is called the frame of discernment framework. Under this premise, the following definitions are provided.

#### **Definition 1**

basic probability assignment function (BPA)^18^.

All subsets of the ${\Theta }$ are denoted as $2^{{\Theta }}$ which represents all possibilities of the proposition to be discriminated. The BPA function (i.e., mass function) is defined as *m*:  $2^{{\Theta }}$ $\in$ [0,1].

1 $$\left\{ {\begin{array}{*{20}l} {\mathop \sum \limits_{{A \subseteq 2^{{\Theta }} }} m\left( A \right) = 1} \hfill \\ {m\left( \emptyset \right) = 0} \hfill \\ \end{array} } \right.$$

This function is also known as the mass function. If *m* (*A*) is greater than 0, *A* is also called a focal element.

#### **Definition 2**

belief and plausibility function^24^.

The belief function is defined as BEL and the formula is as follows:

2 $${\text{BEL}}\left( {\text{A}} \right) = \mathop \sum \limits_{B \subseteq A} m\left( B \right) \left( {\forall A \subset {\Theta }} \right)$$

The belief function refers to the sum of the basic trust probability of all subsets of *A*, where BEL (${\Phi }$) = 0 and BEL (${\Theta }$) = 1. And let PL be the plausibility function, PL(A) = $\mathop \sum \nolimits_{{B \cap A \ne {\Phi }}} m\left( B \right)$. PL(A)-BEL(A) represents the uncertainty of *A*.

#### **Definition 3**

D–S rule^25^.

Let *m*_1_ and *m*_2_ be the two BPA functions on the same discernment framework ${\Theta }$. D–S rule is defined as follows:

3 $$m\left( A \right) = \left\{ {\begin{array}{*{20}l} {0,} \hfill & {A = \emptyset } \hfill \\ {\frac{{\mathop \sum \nolimits_{B \cap C = A} m_{1} \left( B \right)m_{2} \left( C \right)}}{1 - K},} \hfill & {A \ne \emptyset } \hfill \\ \end{array} } \right.$$

where $\forall A \subseteq {\Theta }$, B $\subset {\Theta }$, C $\subset {\Theta }$,${\text{ K}}$ = $\mathop \sum \nolimits_{B \cap C = \emptyset } m_{1} \left( B \right)m_{2} \left( C \right)$. *K* is the conflict between *m*_1_ and *m*_2_. The two pieces of evidence are completely conflict when *K* = 1 and the two pieces of evidence are highly conflict when *K* $\to 1$.

Due to high conflict evidence, the fusion result of the D–S rule may be contrary to common sense. The D–S rule is invalid^26^ when *K* = 1. It is because the denominator is zero in the D–S normalization rule. In addition, the D–S rule failed to address the one-vote veto issue^27^. It means that m(A) is always 0 when the BPA of one piece of evidence is 0, even if much evidence supports A.

### Other combination rules

Aiming to fuse conflicting evidence, Sun et al. measured the average evidence (q) and proposed an effective combination rule based on the evidence credibility $\left( \varepsilon \right)$. Equation (4) shows the evidence credibility function.

4 $$\varepsilon = e^{{ - \frac{1}{{n\left( {n - 1} \right)/2}}\mathop \sum \limits_{i < j} K_{ij} }}$$

where *K*_*ij*_ is the evidence conflict between evidence *i* and *j.* $\frac{1}{{n\left( {n - 1} \right)/2}}\mathop \sum \nolimits_{i < j} K_{ij}$ is the average conflict. When the average conflict increases, the credibility of fusion results decreases.

Here, the improved method in Reference^20^ can be named as KCre-Sun. Equation (5) shows the combination rule.

5 $$m\left( A \right) = \left\{ {\begin{array}{*{20}l} {0,} \hfill & {A = \emptyset } \hfill \\ {p\left( A \right) + K *\varepsilon * q\left( A \right),} \hfill & {A \ne \emptyset ,\Theta } \hfill \\ {p\left( {\Theta } \right) + K* \varepsilon * q\left( {\Theta } \right) + K\left( {1 - \varepsilon } \right),} \hfill & { A = \Theta } \hfill \\ \end{array} } \right.$$

However, the average evidence does not consider the importance of different pieces of evidence, so it is difficult to apply to practical problems. In addition, the evidence credibility $\varepsilon$ in the KCre-Sun method needs to calculate the conflict between any two pieces of evidence, so the calculation complexity is high.

Pan et al. proposed a hybrid combination rule^28^ (namely Hybrid-Rule) to fuse the conflict evidence. When *K* > 0.95, measure the similarity degrees of pieces of evidence by the Euclidean distance in the condition of high evidence conflicts. However, a type of Euclidean distance method cannot measure the complex relationships of pieces of evidence accurately.

## Improved evidence theory

To cope with the counterintuitive fusion results when high conflict pieces of evidence are combined, a lot of work based on the entropy method^29–31^ has been researched to measure the importance of evidence. In addition, credibility^19,20,32^ is measured based on BPAs to represent the evidence divergence. However, the divergence of evidence is sensitive to BPAs^33^, which limits the evidence theory to engineering.

In order to handle the conflict and make reasonable fusion results, this paper introduces the decision credibility to represent the discrepancy of evidence decisions. In addition, the weight of evidence is determined using the entropy weight method. Hence, a weighted combination rule based on decision credibility and evidence weight is presented to meet the engineering field.

1. Decision credibility

Define the pieces of evidence decisions as D = {D_1_, …, D_s_}, ${\Theta } = { }\left\{ {A_{1} ,{ } \ldots ,A_{n} } \right\}$. The evidence decision conflict can be measured by calculating the standard deviation (*d*) of different evidence decisions. The decision credibility is defined as follows:

6 $$\overline{\varepsilon } = \frac{2}{\pi }*{\text{arctan}}\left( \frac{1}{d} \right)$$

where d = $\sqrt {\frac{1}{s}\mathop \sum \limits_{i = 1}^{s} D_{i} - \overline{D}}$. If d = 0, arctan $\left( \frac{1}{d} \right)$ = $\frac{\pi }{2}$. It is because the limits of arctan $\left( \frac{1}{d} \right)$ equals $\frac{\pi }{2}$. Here, $\frac{2}{\pi }$ in Eq. (6) is to make the range of decision credibility [0,1].

(2) Evidence weight

Each evidence contains has the amount of different information. The weights of pieces of evidence can be determined objectively by the entropy weight method. The steps of the entropy weight method are as follows:

*Step 1* The entropy value can be calculated as:

7 $$e_{i} = - 1/log\left( n \right)\mathop \sum \limits_{j = 1}^{n} m_{i} \left( {A_{j} } \right)lnm_{i} \left( {A_{j} } \right)$$

*Step 2* The deviation degree can be calculated as:

8 $$g_{i} = 1 - e_{i}$$

*Step 3* The weights of pieces of evidence can be calculated as:

9 $$w_{i} = \frac{{g_{i} \left( {A_{j} } \right)}}{{\mathop \sum \nolimits_{i = 1}^{s} g_{i} \left( {A_{j} } \right)}}$$

Therefore, based on evidence decision credibility and evidence weight, the combination rule is defined as follows:

10 $$m\left( A \right) = \left\{ {\begin{array}{*{20}l} {0,} \hfill & {A = \emptyset } \hfill \\ {p\left( A \right) + K *\overline{\varepsilon } * w*q\left( A \right),} \hfill & {A \ne \emptyset ,\Theta } \hfill \\ {p\left( {\Theta } \right) + K* \overline{\varepsilon } *w*q\left( A \right) + K\left( {1 - \overline{\varepsilon }} \right),} \hfill & { A = \Theta } \hfill \\ \end{array} } \right.$$

The improved evidence theory in this paper can be named as DCre-Weight method and its algorithm description is shown in Appendix A.

Next, to validate the effectiveness of the proposed algorithm, an example in reference^20^ is introduced to compare the improved evidence theory with the other three combination rules (seen in Part 2.2). Table 1 shows the fusion results of the four combination rules.

**Table 1:** Comparison of the fusion process under the four combination rules.

Methods	Combination	A	B	C	${\Theta }$	Credibility
D–S	*m*_1_ $\oplus$ *m*_2_	0	0.010 0	0.990 0	0	None
	*m*_1_ $\oplus$ *m*_2_ $\oplus$ *m*_3_	0	0	1	0	None
KCre-Sun	*m*_1_ $\oplus$ *m*_2_	0.180 0	0.004 0	0.194 0	0.622 0	0.3716
	*m*_1_ $\oplus$ *m*_2_ $\oplus$ *m*_3_	0.321 0	0.003 0	0.188 0	0.488 0	0.5120
Hybrid-Rule	*m*_1_ $\oplus$ *m*_2_	0.490 0	0.010 0	0.500 0	0	None
	*m*_1_ $\oplus$ *m*_2_ $\oplus$ *m*_3_	0.737 5	0.005 9	0.256 6	0	None
DCre-Weight	*m*_1_ $\oplus$ *m*_2_	0.400 7	0.008 5	0.436 5	0.154 4	0.8440
	*m*_1_ $\oplus$ *m*_2_ $\oplus$ *m*_3_	0.529 4	0.006 3	0.345 9	0.118 5	0.8814

### ***Example 1***

There are three pieces of evidence, m_1_, m_2_, and m_3_. The initial BPA values of the three evidences on the target A, B and C are as follows: *m*_1_: *m*_1_(*A*) = 0.98, *m*_1_(*B*) = 0.01, *m*_1_(*C*) = 0.01; *m*_2_: *m*_2_(*A*) = 0, m_2_(*B*) = 0.01, *m*_2_(*C*) = 0.99; *m*_3_: *m*_3_(*A*) = 0.9, *m*_3_(*B*) = 0, *m*_3_(*C*) = 0.1.

According to the results in Table 1, it failed to recognize target A by D–S evidence theory because of the conflict evidence m_2_. Target A is recognized correctly using the KCre-Sun and Hybrid-Rule methods. However, in the fusion process, the credibility of target A is low using the KCre-Sun method. Compared with the KCre-Sun method, the proposed DCre-Weight method and the Hybrid-Rule method improved the credibility of fusion results. However, m (${\Theta }$) is always 0 in the fusion of *m*_1_ $\oplus$ *m*_2_ and *m*_1_ $\oplus$ *m*_2_ $\oplus$ *m*_3_ using the Hybrid-Rule method. It cannot express the uncertainty in the combined decision. Compared with the Hybrid-Rule, because the proposed method measured the decision credibility by calculating the difference of evidence decisions and assigned the evidence conflict according to the evidence weight, the value of m (Θ) is decreased when the third piece of evidence is combined.

## Ambient air quality evaluation model

Nowadays, air quality data can be easily accumulated by sensors around the world^34^. The concentration of pollutants monitored at monitoring stations changes with meteorological conditions, policies, pollution sources, human factors, etc. Evidence theory can well address the ambiguity of air quality and the uncertainty of environmental systems. For air quality evaluation, the main air pollutants affecting air quality are CO, PM_10_, NO_2_, PM_2.5_, SO_2_, O_3_. Air quality is not determined by a single air pollutant, but a combination of multiple air pollutants. Through the fusion pollution information through the improved evidence theory, a more accurate assessment of air quality can be obtained.

**Figure 1:**
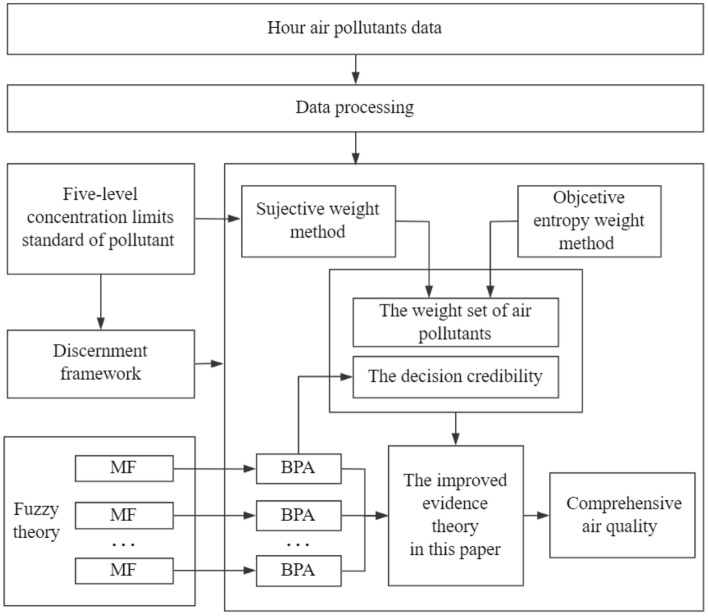
Air quality evaluation model based on improved evidence theory.

The air quality evaluation model based on the improved evidence theory is shown in Fig. 1. Firstly, the membership functions (MFs)^35^ of each air pollutant are established based on fuzzy theory and transformed into BPA functions. Then the weight set of pollutants is established according to the evaluation standard and entropy weight method. Finally, the improved evidence theory is used to fuse the information of multiple pollutants.

### Evaluation standards

The AQI standards for China and the United States are the same, but the concentration limits of pollutants are different, especially the limits of PM_2.5_. According to the standard AQI (HJ633-2012[Z]) and the Ambient Air Quality Standards (GB 3095-2012), this paper revised the limits of some pollutants and established five criterion levels, as shown in Table 2.

**Table 2:** Air quality standards.

Pollutant	Level I	Level II	Level III	Level IV	Level V
SO_2_	50	125	250	350	450
NO_2_	40	80	120	160	200
CO	2	4	6	8	10
O_3_	100	160	200	265	320
PM_10_	50	150	250	350	420
PM_2.5_	25	75	115	150	250

Air pollutants have impacts on human respiratory system. The description of the air quality evaluation standard is shown in Table 3.

**Table 3:** Description of air quality standards.

Air Pollution Level	Impact on health	Suggestions
Level I (Good)	Little impact on health	Enjoy outdoor activities
Level II (Regular)	Weak impact on health	Sensitive groups should reduce prolonged outdoor activities
Level III (Light Pollution)	Minor impact on health. It may irritate the respiratory tract	Susceptible people reduce prolonged outdoor activities
Level IV (Moderate Pollution)	Greater impact on health. It may exacerbate chronic bronchitis disease	Healthy people should reduce prolonged outdoor activities and the other people should restrict outdoor activities
Level V (Heavy Pollution)	Extremely harmful to health. It may cause difficulty breathing and chest tightness. And it may hurt the eyes	Healthy people should restrict outdoor activities and the other people should remain indoors

### Determining the membership functions (MFs)

The events in the discernment framework ${\Theta }$ are regarded as fuzzy sets {*A*_1_, …, *A*_*n*_} of the domain *U*. The membership degree of the object is transformed into the BPA using the normalization method.

Set *U* = {I, II, III, IV, V,${\Theta }$} and define *s* air pollutants as the indicators set. According to the characteristics of pollutants in Table 2, the MFs are built for any recognition object *x*_*i*_ in *X* = {*x*_1_, …, *x*_*s*_}. When the concentration of pollutants, *x*_*i*_, exceeds the limit of level *j*-1, the degree of membership of the previous quality level *j*-1 decreases, and the degree of membership of the next level *j* + 1 increases. But the change between air quality and pollutant concentration is non-linear. Let $y_{ij}$ be the concentration limit of the quality level *j* of *x*_*i*_. Here, the increasing function uses $\log_{2} \left( {1 + \frac{{x_{i} }}{{y_{{i\left( {j - 1} \right)}} }}} \right)$ instead of the linear function $\frac{{x_{i} }}{{y_{{i\left( {j - 1} \right)}} }}$. The decreasing function uses $\left( {\frac{{y_{{i\left( {j + 1} \right)}} - x_{i} }}{{y_{{i\left( {j + 1} \right)}} - y_{ij} }}} \right)^{2}$ instead of the $\frac{{y_{{i\left( {j + 1} \right)}} - x_{i} }}{{y_{{i\left( {j + 1} \right)}} - y_{ij} }}$ linear function.

If the concentration of some pollutant is less than the limit of first-level, the air quality is judged as level 1, and the membership function is improved from the Z function, as shown in Eq. (11). If the concentration of some pollutant exceeds the limit of level *j*-1, where 2 $\le j \le 4$, the quality is judged as level *j*, and Eq. (12) is selected. If the concentration of some pollutant is over the limit of level 4, it is judged as level 5, and Eq. (13) is selected. The MFs of each air pollutant related to the five criterion levels can be selected as follows:

Level I, *j* = 1

11 $$u_{ij} = \left\{ {\begin{array}{*{20}l} {1,} \hfill & {x_{i} \le y_{ij} } \hfill \\ {\left( {\frac{{y_{{i\left( {j + 1} \right)}} - x_{i} }}{{y_{{i\left( {j + 1} \right)}} - y_{ij} }}} \right)^{2} } \hfill & {y_{ij} < x_{i} \le y_{{i\left( {j + 1} \right)}} } \hfill \\ {0,} \hfill & {x_{i} > y_{{i\left( {j + 1} \right)}} } \hfill \\ \end{array} } \right.$$

Level II to level IV, *j* = 2, 3, 4

12 $$u_{ij} = \left\{ {\begin{array}{*{20}l} {\log_{2} \left( {1 + \frac{{x_{i} }}{{y_{{i\left( {j - 1} \right)}} }}} \right),} \hfill & {x_{i} \le y_{{i\left( {j - 1} \right)}} } \hfill \\ {1,} \hfill & {y_{{i\left( {j - 1} \right)}} < x_{i} \le y_{ij} } \hfill \\ {\left( {\frac{{y_{{i\left( {j + 1} \right)}} - x_{i} }}{{y_{{i\left( {j + 1} \right)}} - y_{ij} }}} \right)^{2} ,} \hfill & {y_{ij} < x_{i} \le y_{{i\left( {j + 1} \right)}} } \hfill \\ {0,} \hfill & {x_{i} > y_{{i\left( {j + 1} \right)}} } \hfill \\ \end{array} } \right.$$

Level V, *j* = 5

13 $$u_{ij} = \left\{ {\begin{array}{*{20}l} {\log_{2} \left( {1 + \frac{{x_{i} }}{{y_{{i\left( {j - 1} \right)}} }}} \right),} \hfill & {x_{i} \le y_{{i\left( {j - 1} \right)}} } \hfill \\ {1,} \hfill & {x > y_{{i\left( {j - 1} \right)}} } \hfill \\ \end{array} } \right.$$

where *i* = 1, …, *m*, and *j* = 1, …, *n*. The membership of indicators belonging to each mode is shown in Eq. (14).

14 $$\left[ {\begin{array}{*{20}l} {u_{{1A_{1} }} \left( x \right)} \hfill & {u_{{1A_{2} }} \left( x \right)} \hfill & \ldots \hfill & {u_{{1A_{n + 1} }} \left( x \right)} \hfill \\ {u_{{2A_{1} }} \left( x \right)} \hfill & {u_{{2A_{2} }} \left( x \right)} \hfill & \ldots \hfill & {u_{{2A_{n + 1} }} \left( x \right)} \hfill \\ \ldots \hfill & \ldots \hfill & \ldots \hfill & \ldots \hfill \\ {u_{{sA_{1} }} \left( x \right)} \hfill & {u_{{sA_{2} }} \left( x \right)} \hfill & \ldots \hfill & {u_{{sA_{n + 1} }} \left( x \right)} \hfill \\ \end{array} } \right]$$

In this study, the evidence theory is applied to the evaluation model of ambient air quality. The first step is the initial belief probability in the model. Since the mass function in D–S theory represents the basic trust of a certain proposition A, and the degree of membership represents the degree that the object belongs to the fuzzy sets, the mass function can be transformed by the membership function. The mass functions of object *x* can be calculated by Eq. (15).

15 $$m_{i} \left( {A_{j} } \right) = \frac{{u_{{iA_{j} }} \left( x \right)}}{{\mathop \sum \nolimits_{j = 1}^{n + 1} u_{{iA_{j} }} \left( x \right)}},i = {1},{ 2}, \ldots ,s;j = {1},{ 2}, \ldots ,n + {1}$$

### Air quality evaluation based on improved evidence theory

Based on the improved evidence theory (DCre-Weight), the air quality model (DCreWeight) is proposed to evaluate comprehensive air quality. Firstly, considering the contributions of pollutant to air quality evaluation, the weights of pollutants are built based on the subjective weight method and the objective entropy weight method. Then, define the concentration of six air pollutants as pieces of evidence and use the improved evidence theory to make a comprehensive decision of air quality level.

The steps of the DCreWeight model are as follows:

1. Set *U* = {I, II, III, IV, V, ${\Theta }$}. I, II, III, IV, V means the air quality levels, and ${\Theta }$ represents the uncertainty in air quality evaluation.
2. According to the MFs in Equal (11), Equal (12), and Equal (13), the BPA can be established.
3. Standardize the evaluation data $(x_{ij} )_{m \times n}$ according to Eq. (16). And calculate the ration $p_{ij} = x^{\prime}_{ij} /\mathop \sum \nolimits_{i = 1}^{m} x^{\prime}_{ij}$. Then the weights of air pollutants (W_1_) can be calculated according to the entropy weight method in Eq. (7) ~ Eq. (10).
  16 $$x_{ij}^{\prime } = (\max \left( {x_{ij} , \ldots ,x_{ij} } \right) - x_{ij} )/\left( {\max \left( {x_{ij} , \ldots ,x_{ij} } \right) - \min \left( {x_{ij} , \ldots ,x_{ij} } \right)} \right)$$
4. Using the subjective weight method to establish the weights of air pollutants. The excessive times method is as follows.
  17 $$a_{i} = \left( {j - 1} \right) + \frac{{x_{i} - y_{{i\left( {j - 1} \right)}} }}{{y_{ij} - y_{{i\left( {j - 1} \right)}} }},i = {1},{ 2}, \ldots ,s,j = {1},{ 2},{ 3}, \ldots ,n$$
  where $y_{ij}$ is the limits of pollutants in Table 2 and $x_{i}$ is the real concentration of pollutant *i*. If j = 1, $y_{i0} = 0$. Particularly, when the weight exceeds the *n*^th^ level of concentration limit, $a_{i} = j + \frac{{x_{i} }}{{y_{ij} }}$.
  Define the normalized weights as W_2_. Establish appropriate weights {a, b} for w_1_ and w_2_. Then the weight set of evidence is W=a*W_1_+b*W_2_. Here set the {a, b} = {0.2, 0.8} to the highlight the impacts of main pollutants on air quality.
5. Accoding to Eq. (10) in the DCre-Weight method, using the proposed combiantion rule to to evlaute the comprehensive air quality. The obtained probabilities are shown as Eq. (18).
  18 $$P\, = \,\{ {\text{P}}\left( {\text{I}} \right),P\left( {{\text{II}}} \right),P\left( {{\text{III}}} \right),P\left( {{\text{IV}}} \right),P\left( {\text{V}} \right),P(\Theta )\}$$
6. According to the maximum probability, the comprehensive air quality (Level) can be determinded according to Eq. (19).
  19 $${\text{Level}}\, = \,max\{ {\text{P}}\}$$

### A case of air evaluation based on improved evidence theory

To state the application of the model, we take Example 2 to compare the air quality model based on the DCre-Weight algorithm with other combination rules of evidence theory, as shown in Table 4.

**Table 4:** Comparison with air quality evaluation methods using evidence combination rules.

Methods	I	II	III	IV	V	${\Theta }$	Credibility	Result
D–S	0	0	0	0.0043	0.9957	0	None	V
KCre-Sun	0.0762	0.0687	0.0687	0.0596	0.1743	0.5533	0.4467	V
Hybrid-Rule	0.1653	0.1169	0.0787	0.0766	0.5626	0	None	V
DCre-Weight	0.1121	0.0831	0.0958	0.1128	0.5336	0.0625	0.9647	V

#### ***Example 2***

There are mainly six pollutants that affect air quality. Take a piece of data as an example to analyze the comprehensive air quality level. SO_2_ = 46, NO_2_ = 74, CO = 4.96, O_3_ = 16, PM_10_ = 390, PM_2.5_ = 241 in Xi'an on January 5, 2016.

According to the maximum probability, the comprehensive air quality level is V and the air quality is most likely to be heavily polluted by the above methods given in Table 4. Compare to the D–S and Hybrid-Rule, the uncertainty is not 0 by the KCre-Sun and DCre-Weight method due to different pollution degrees of six pollutants. However, the level V is only 0.1743 and the credibility is 0.4467 by the KCre-Sun method. Compared to the KCre-Sun method, the fusion results contain more useful information by DCre-Weight, which is conducive to decision-making.

## Results

### Data

**Figure 2:**
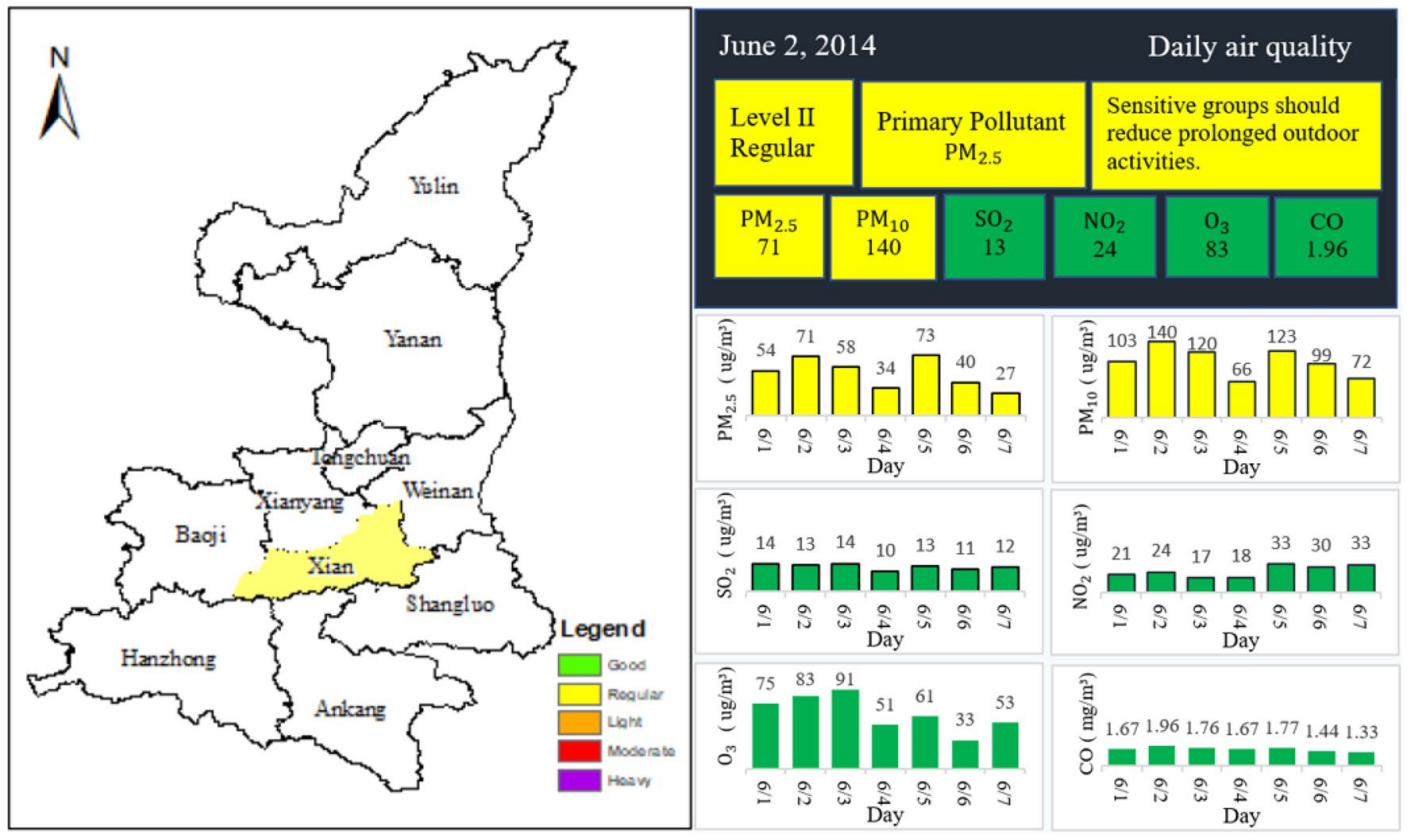
Air quality in Xi’an on June 2, 2014. A map of the Shaanxi province of China. (Generated by ArcGIS 10.8, URL: http://www.esri.com/software/arcgis/arcgis-for-desktop).

To validate the performance of the proposed DCreWeight model, select hourly air pollution data in Xi’an from June 1, 2014, to May 1, 2016. The years are randomly selected. In this paper, the null values are processed using the linear interpolation method. According to the proposed DCreWeight model, the comprehensive air quality evaluation results on a day are as follows (see Fig. 2).

### Evaluation indicators

1. Evaluation indicators based on AQI

The national AQI standard (HJ633-2012[Z]) describes the air quality level. AQI standard denotes that the highest pollutant concentration determines the air quality level. It highlights the contribution of one pollutant. Equation (20) shows the calculation of AQI. It defines the concentration limits [BP_Lo_, BP_Hi_] and IAQI limits [IAQI_Hi_, IAQI_Lo_].

20 $${\text{AQI}} = {\text{max }}\left( {\left( {{\text{IAQI}}_{{{\text{Hi}}}} - {\text{ IAQI}}_{{{\text{Lo}}}} } \right)*\left( {{\text{C}}_{{\text{P}}} - {\text{BP}}_{{{\text{Lo}}}} } \right)/\left( {{\text{BP}}_{{{\text{Hi}}}} - {\text{BP}}_{{{\text{Lo}}}} } \right) + {\text{IAQI}}_{{{\text{Lo}}}} } \right)$$

where C_P_ is the concentration of pollutant P.

Taking the national AQI as the pollution standard, the indicator MAE, RMSE and an index of agreement can be calculated to analyze the performance of evaluation models. Count the number of days when AQI is equal to the evaluation level of models, and define it as right_num.

Defined AQI_MAE, AQI_RMSE and AQI_an index of agreement as evaluation indicators. The above evaluation indicators based on AQI can be calculated as follows:

21 $${\text{AQI}}\_{\text{MAE}} = \frac{1}{n}\mathop \sum \limits_{i = 1}^{n} \left( {h_{i} - y_{i} } \right)$$

22 $${\text{AQI}}\_{\text{RMSE}} = \sqrt {\frac{1}{n}\mathop \sum \limits_{i = 1}^{n} \left( {h_{i} - y_{i} } \right)^{2} }$$

23 $${\text{AQI}}\_{\text{an}}\;{\text{index}}\;{\text{of}}\;{\text{agreement}} = \frac{{{\text{right}}\_{\text{num}}}}{n}$$

where n is the number of samples, $y_{i}$ is the actual AQI value of the i-th day, $h_{i}$ is the evaluation result of a model.

(2) Evaluation indicators based on AQCI

The national AQCI considers the comprehensive impacts of multiple pollutants on air quality. It highlights the contribution of six pollutants. AQCI is shown in Eq. (24).

24 $${\text{AQCI}} = {\text{sum }}\left( {{\text{C}}_{{\text{P}}} /{\text{S}}_{{\text{P}}} } \right)$$

where S_P_ is the second concentration limit of pollutant P in the Ambient Air Quality Standards (GB 3095-2012).

Taking the national AQCI as the pollution standard, the indicator AQCI_MAE and AQCI_RMSE can be calculated by Eqs. (21) and (22) in the same way.

### Analysis and comparison of evaluation methods

Take national AQI and AQCI as pollution standards. The comparisons of the DCreWeight model with the D–S, KCre-Sun, Hybrid-Rule, and FSE models are in Fig. 4. For the clarity of the image, select four months from June 1, 2014 to March 31, 2015, which can roughly represent four seasons. Spring is represented by March. Summer represented by June. Autumn is represented by September. Winter is represented by December.

**Figure 3:**
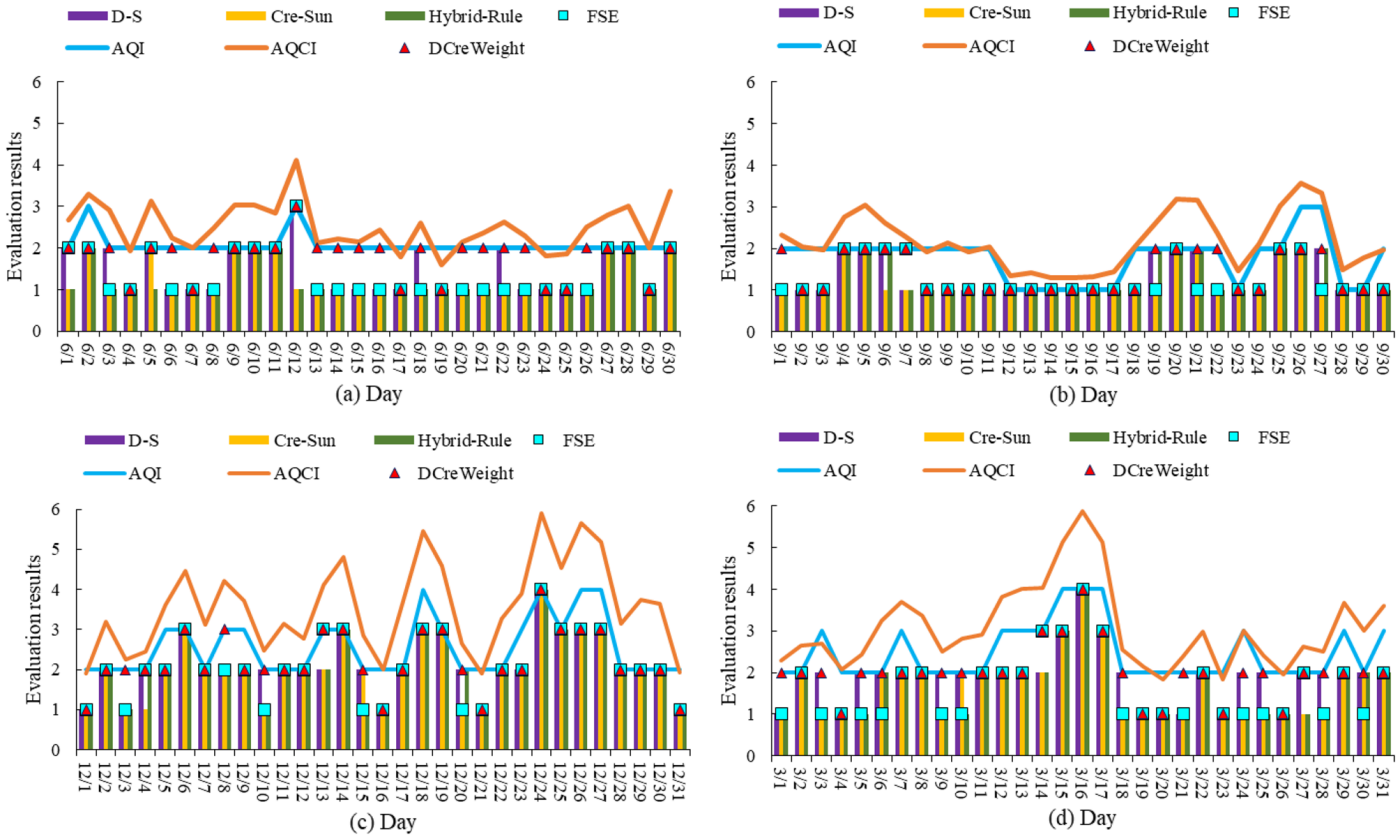
Daily air quality in Xi’an in four seasons. (**a**) Daily air quality in Summer; (**b**) daily air quality in Autumn (**c**) daily air quality in Winter; (**d**) daily air quality in Spring.

**Figure 4:**
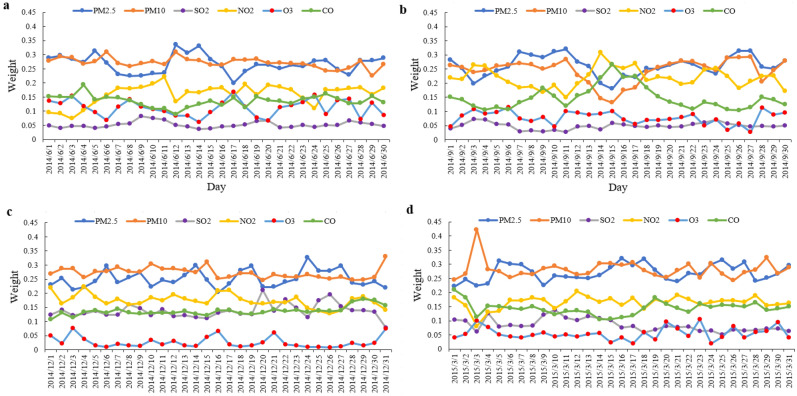
Weight of pollutants in different seasons. (**a**) Weight of pollutants in summer; (**b**) weight of pollutants in autumn; (**c**) weight of pollutants in winter; (**d**) weight of pollutants in spring.

According to Figs. 3 and 4, the air pollution situations were Winter > Spring > Summer > Autumn. PM_2.5_ and PM_10_ were primary pollutants in the four months. In Winter, the weight of SO_2_ was greater than that of O_3_. But in the other three months, it was smaller than that of O_3_. It is because that the weak light made O_3_ concentration decreased, and coal burning for heating made an increase of SO_2_ in Winter. It is because that the weak light reduces the O_3_ concentration while coal burning for heating increases SO_2_ concentration in Winter. Take national AQI and AQCI as pollution standards, the evaluated air quality levels of D–S, KCre-Sun, Hybrid-Rule, and FSE methods are mostly lower than AQI. The evaluated results of the above models deviate greatly from the AQI and AQCI, while the evaluated results of the DcreWeight model are closest to the national AQI and AQCI.

**Figure 5:**
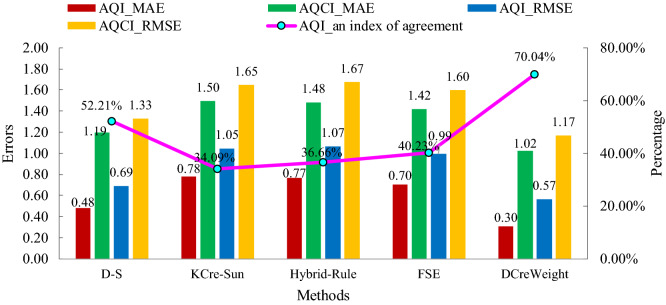
Performance comparison results of the evaluation methods.

To validate the superiority of the models, take AQI_MAE, AQI_RMSE, AQI_an index of agreement, AQCI_MAE, and AQCI_RMSE as evaluation indicators. The performance comparison results of the evaluation methods under the AQI and AQCI standards are shown in Fig. 5.

According to Fig. 5, the DCreWeight model has the minimum MAE, RMSE under the AQI and AQCI standards and its index of agreement is the highest, which is superior to the D–S, KCre-Sun, Hybrid-Rule, and FSE methods.

### The application in Shanghai and Beijing

The superiority of the model has been validated according to air pollutants data in Xi'an in “Analysis and comparison of evaluation methods” section. In order to better check whether the model is suitable for other urban air quality assessments, we also selected hourly air pollution data from 2014 from June 1, 2014, to May 31, 2015, in Shanghai and Beijing. Firstly, the null data were processed using the linear interpolation method. Then, we applied the DCreWeight model to the two cities and compared the air quality between Shanghai and Beijing under the national AQI and AQCI standards.

**Figure 6:**
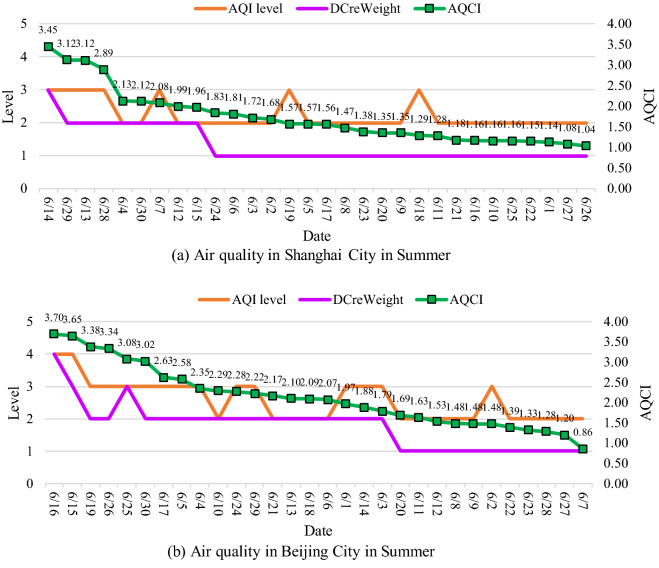
Air quality in Shanghai and Beijing City in Summer from June 1 to June 30.

Figure 6 shows the evaluation results of the DCreWeight model in Summer, from June 1, 2014, to June 31, 2014. The left vertical axis represents the air quality evaluation level, and the right vertical axis represents the AQCI value. National AQCI represents the comprehensive pollution degree. To clearly check the accuracy of the DCreWeight model, sort the days according to AQCI.

According to Fig. 6, the AQI level fluctuates as the AQCI value decreases. This is because the AQI level depends on individual pollutants. However, with the decrease of AQCI, the evaluation results of the DCreWeight model basically decline in steps. It indicates that the evaluation of the model is in line with the actual comprehensive pollution. Compared with AQCI, the proposed model describes air quality levels more intuitively.

**Figure 7:**
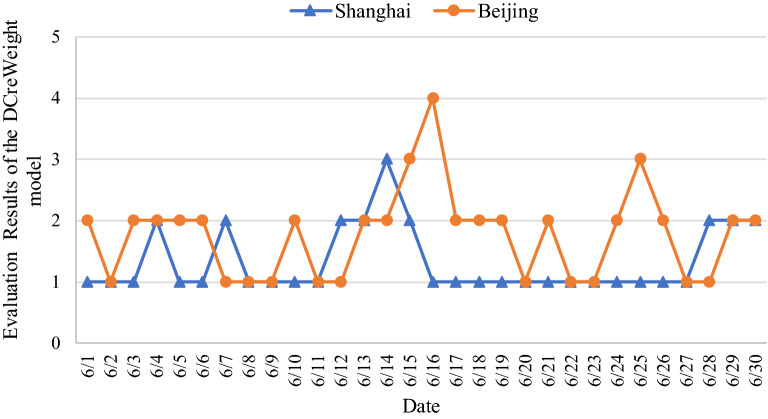
Evaluation Results of the DCreWeight model.

Next, compare the air quality between Shanghai and Beijing, as shown in Fig. 7.

According to Fig. 7, the following conclusions can be drawn. The air quality in Beijing in Summer was worse than that in Shanghai. The comprehensive air quality is good or regular basically in Shanghai in Summer. However, many days are regular, lightly polluted, and moderately polluted in Beijing in Summer.

**Figure 8:**
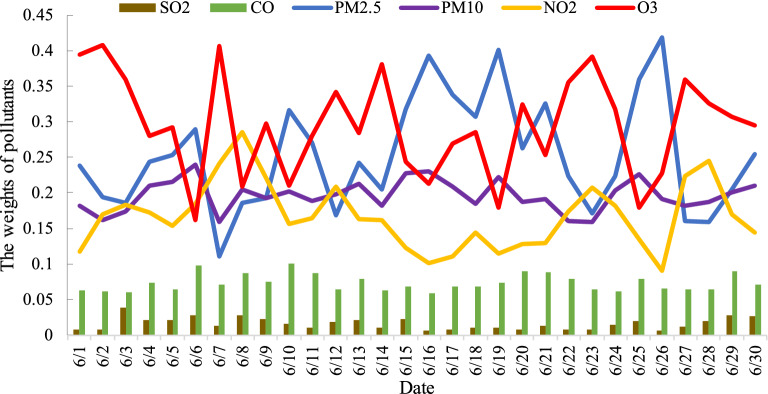
The weights of pollutants in Beijing.

Finally, given that pollution control is a long-term process, we finally analyze the pollution characteristics in Beijing according to the weights of pollutants, as is shown in Fig. 8. We also analyze the possible reasons for pollution to help relevant departments make strong pollution strategies based on pollution characteristics and the current air quality level.

We can conclude that PM_2.5_, O_3_, and PM_10_ are the main pollutants in Beijing according to Fig. 8. There are many causes of PM_2.5_ in Beijing, including vehicle exhaust, industrial emissions, and dust from construction sites and road traffic, all of which increase the concentration of PM_2.5_. In summer, under the strong ultraviolet light, nitrogen oxides are more easily converted into O_3_ by photochemical reaction, so the concentration of O_3_ will increase. The exposed arable land around Beijing and the surrounding sandy areas, as well as the monsoon climate in Beijing, will cause PM_10_ concentration to increase under the wind effect. In addition, vehicle exhaust and industrial waste gases also cause a higher concentration of NO_2_. Therefore, although NO_2_ concentration is not as high as PM_2.5_, O_3_, and PM_10_ pollution, we should pay attention to NO_2_ concentration control in summer to avoid a high O_3_ concentration. The relevant departments should control air pollution from the sources of traffic, industry park, and construction sites, and protect the surrounding environment by planting green plants.

## Conclusion

Air quality is affected by many air pollutants. Selecting appropriate methods to evaluate air quality is the basis for taking relevant air pollution control measures. This paper proposed an air quality evaluation model based on the improved evidence theory. The core part of the model is to use the improved evidence theory (DCre-Weight) to evaluate the comprehensive impact of multiple air pollutants on air quality. An algorithm case showed that the DCre-Weight method improved the credibility of fusion results, which solved the counterintuitive fusion results in D–S evidence theory. And the uncertainty was well expressed using the DCre-Weight method. In addition, a specific application of this model in Xi’an shows that the DCreWeight model comprehensively evaluates air quality. Under the national AQI and AQCI as pollution standards, the MAE and RMSE values of the proposed model were minimal and the index of agreement was maximal, which validated the superiority of the DCreWeight model.

Air quality is closely related to human life and air quality evaluation is of great value and significance to the ecological environment. This paper considers the influence of multiple pollutants and comprehensively evaluated daily air quality, which is a supplement to the AQI evaluation method. The limitation of this method is that it may not be applicable in special high pollution areas. The air quality comprehensive evaluation model based on improved evidence theory can be applied to tourism industry and government departments. It can provide a reference for the tourism and support for the government in assessing air quality and developing long-term pollution prevention and control strategies. However, this paper studies the comprehensive evaluation of air quality based on existing hourly concentration of pollutants. It does not involve the prediction of pollutant concentrations. In our future research, the concentration of six pollutants prediction will be conducted. Then an air quality prediction and evaluation model will be established to form a relatively complete air quality research. In addition, with the increase of air pollution monitoring points, real-time monitoring data has surged. Therefore, the data processing and data fusion method are also the keys to assessing air quality accurately.

## Supplementary Information

Supplementary Information.
