# Supplementary material for: An ambient air quality evaluation model based on improved evidence theory
Source: Sci Rep. 2022 Apr 6;12:5753. doi: 10.1038/s41598-022-09344-0 (PMC8986843; doi:10.1038/s41598-022-09344-0)
Supplement: Supplementary file 1 — Supplementary Information. [file 41598_2022_9344_MOESM1_ESM.docx]

# Appendix A

| **Algorithm 1**：DCre-Weight algorithm  **Input:** mass functions *m= {m*_1_*, …, m_s_}*  **Output:** the fusion results and the credibility of fusion results |
| --- |
| 1. [n, s] =size(m);   % The weight of pieces of evidence are calculated by the entropy weight method.   1. e=-1/log(n).*sum(m,1); 2. g=1-e; 3. w=g./sum(g,2);   % The dispersion of different evidence decisions is determined by calculating the standard deviation.   1. bpa=m.’ 2. [~, decisions] =max (bpa, [], 2); 3. normDecisions= decisions./ sum(decisions,1); 4. avgDeci =sum(normDecisions,1)./s; 5. var=sum(power((normDecisions-avgDeci),2),1)/s; 6. d=sqrt(var);   % Decision credibility   1. crd=2/pi*atan(1/d);   % Combine the pieces of evidence   1. p = bpa (1, :) 2. uncertain=bpa(:,n); 3. **for** i=1: s-1 4. p =p.* bpa(i+1,:)+ p(1,n).*bpa(i+1,:)+ bpa(i+1,n) .* p(1,:); 5. px= p(1,n)*bpa(i+1,n); 6. p(1,n)=px; 7. k=1-sum(p,2); 8. **end** 9. p= p+k*crd.*(w*bpa); 10. px = px +k*crd.*(w*bpa(:,n))+k*(1-crd); 11. p(1,n)=px; 12. results=p; |
